# Patent foramen ovale closure vs. medical therapy for cryptogenic stroke: a meta-analysis of randomized controlled trials

**DOI:** 10.1093/eurheartj/ehy121

**Published:** 2018-03-24

**Authors:** Yousif Ahmad, James P Howard, Ahran Arnold, Matthew Shun Shin, Christopher Cook, Ricardo Petraco, Ozan Demir, Luke Williams, Juan F Iglesias, Nilesh Sutaria, Iqbal Malik, Justin Davies, Jamil Mayet, Darrel Francis, Sayan Sen

**Affiliations:** National Heart and Lung Institute, 2nd Floor B Block, Hammersmith Hospital, Imperial College London W12 0HS, UK

**Keywords:** PFO, Closure, Patent foramen ovale, Cardiology, Interventional, Cryptogenic • Stroke

## Abstract

**Aims:**

The efficacy of patent foramen ovale (PFO) closure for cryptogenic stroke has been controversial. We undertook a meta-analysis of randomized controlled trials (RCTs) comparing device closure with medical therapy to prevent recurrent stroke for patients with PFO.

**Methods and results:**

We systematically identified all RCTs comparing device closure to medical therapy for cryptogenic stroke in patients with PFO. The primary efficacy endpoint was recurrent stroke, analysed on an intention-to-treat basis. The primary safety endpoint was new onset atrial fibrillation (AF). Five studies (3440 patients) were included. In all, 1829 patients were randomized to device closure and 1611 to medical therapy. Across all patients, PFO closure was superior to medical therapy for prevention of stroke [hazard ratio (HR) 0.32, 95% confidence interval (95% CI) 0.13–0.82; *P* = 0.018, *I*^2^ = 73.4%]. The risk of AF was significantly increased with device closure [risk ratio (RR) 4.68, 95% CI 2.19–10.00, *P*<0.001, heterogeneity *I*^2^ = 27.5%)]. In patients with large shunts, PFO closure was associated with a significant reduction in stroke (HR 0.33, 95% CI 0.16–0.72; *P* = 0.005), whilst there was no significant reduction in stroke in patients with a small shunt (HR 0.90, 95% CI 0.50–1.60; *P* = 0.712). There was no effect from the presence or absence of an atrial septal aneurysm on outcomes (*P* = 0.994).

**Conclusion:**

In selected patients with cryptogenic stroke, PFO closure is superior to medical therapy for the prevention of further stroke: this is particularly true for patients with moderate-to-large shunts. Guidelines should be updated to reflect this.

## Introduction

The incidence of stroke in the United States is approximately 800 000 per year, 30% of which are cryptogenic.^1^ In up to 40% of these patients, a patent foramen ovale (PFO) is present.^2^ In the presence of a PFO, a clot in the venous circulation can travel across the PFO and lead to arterial occlusion; this paradoxical embolism can lead to a stroke. Subclinical episodes of atrial fibrillation (AF) could be a further mechanism for stroke.

Observational studies have suggested an association between the presence of PFO and cryptogenic stroke.^3^ Percutaneous closure of PFOs using catheter-based systems has been available since the 1990 s. The superiority of device closure over medical therapy for the prevention of recurrent strokes in patients with cryptogenic stroke and a PFO has not been established. Three previously published randomized controlled trials (RCTs) did not demonstrate superiority of device closure over medical therapy.^4–6^ Meta-analyses of these trials have also not conclusively proven any significant benefit of device closure when analysed on an intention-to-treat basis.^7^^,^^8^

Existing guidelines, written before recent trial data was available, do not recommend routine closure of PFOs for patients with cryptogenic stroke. Advisory committees have recommended restricting the closure of PFOs to ongoing clinical trials. Nevertheless, many patients continue to be treated ‘off-label’.^9^

Two recent RCTs comparing PFO closure to medical therapy have been published,^10^^,^^11^ along with updated long-term results from a previous RCT.^12^ We therefore conducted a meta-analysis of RCT data including the most recent trials to formally evaluate the benefit of percutaneous closure of a patent foramen ovale after a cryptogenic stroke.

## Methods

We carried out a meta-analysis of RCTs that evaluated device closure for patients with a PFO and cryptogenic stroke.

### Search strategy

We performed a systematic search of the MEDLINE, Cochrane Central Register of Controlled Trials, and Embase databases from October 2000 to October 2017 for all studies of PFO closure. Our search strings included ‘(PFO or “patent foramen ovale”) AND Closure’; and ‘cryptogenic stroke’, respectively. We also hand-searched the bibliographies of relevant selected studies, reviews and meta-analyses to identify further eligible studies. Abstracts were reviewed for suitability and articles accordingly retrieved. Two independent reviewers performed the search and literature screening (Y.A. and M.S.S.), with disputes resolved by consensus following discussion with a third author (S.S.).

### Inclusion and exclusion criteria

We considered all randomized studies of PFO closure. Studies were eligible if they randomized patients to device closure or medical therapy, and reported outcome data with regards to recurrent stroke. Observational studies were not considered.

### Endpoints

The primary efficacy endpoint was recurrent stroke and the primary safety endpoint was risk of AF. Non-fatal and fatal ischaemic strokes were included together as stroke in endpoint definitions across trials. We considered major bleeding as a secondary safety endpoint.

### Data extraction and analysis

Two authors (Y.A. and A.A.) independently abstracted the data from included trials, verified by a third author (J.H.). We analysed efficacy on an intention-to-treat basis. The primary outcome measure was the hazard ratio (HR) of recurrence of stroke.

We extracted the HRs with their associated 95% confidence intervals (95% CI) and *P*-values. A random-effects meta-analysis was performed of the natural logarithm of the HRs and their associated standard errors using the restricted maximum likelihood (REML) estimator. The standard error was calculated by dividing the difference between the natural logarithms of the upper and lower 95% CIs by 2× the appropriate normal score (1.96). Where the lower 95% CI approached zero, the standard error was calculated using only the difference between the natural logarithm of the upper 95% CI and the natural logarithm of the point estimate. Interactions between subgroups were assessed using a mixed effects meta-analytical model, with both the trial and subgroup in question as moderators. We used the *I*^2^ statistic to assess heterogeneity.^13^ Mean values are expressed as mean ± SD unless otherwise stated. The statistical programming environment R^14^ with the metafor package^15^ was used for all statistical analysis.

Included studies were assessed using the Cochrane Risk of Bias tool.^16^ Tests for publication bias would only be performed in the event of at least 10 trials being included for analysis.^17^

Results were reported in accordance with the PRISMA guideline.^18^

### Subgroups

We specified the size of shunt and presence of an aneurysmal atrial septum as subgroup analyses. The definition of small shunt, where stated, was less than 10 microbubbles seen in the left atrium on bubble study in all trials. The definition of substantial/large shunt varied, where stated. Therefore, we compared moderate-large shunt to small shunt.

## Results

**Figure 1:**
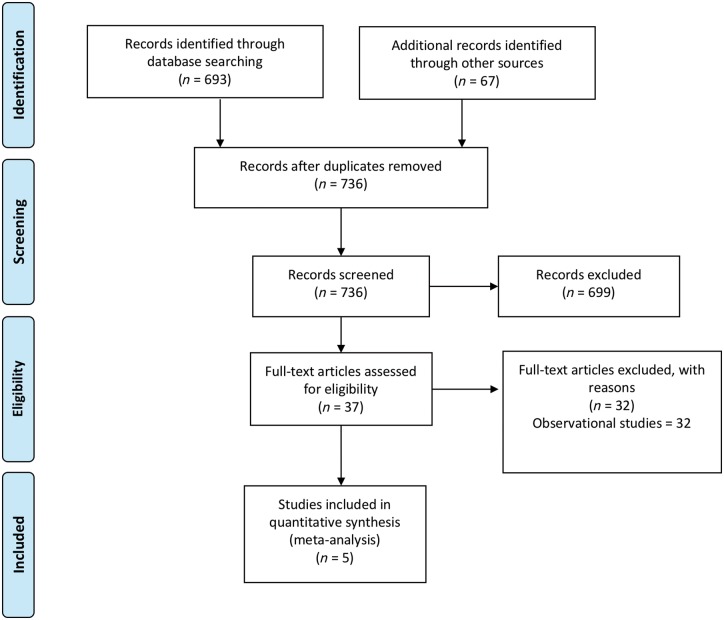
Search strategy and source of included studies.

Five studies,^4^^,^^6^^,^^10–12^ enrolling 3440 patients met the inclusion criteria (*Figure 1*). In all, 1829 patients were randomized to device closure and 1611 to medical therapy, with a weighted mean follow-up of 4.01 years in the device group and 4.07 years in the medical therapy group. Across the five studies, the mean age was 44.9 years. The full characteristics of included studies are shown in *Table 1*. All trials enrolled patients with a prior ischaemic stroke and no identifiable cause apart from a PFO; three specified within the prior 6 months and one within the prior 9 months, with one trial not specifying a timescale. The primary endpoint varied across trials including ischaemic stroke in one trial, and various composites of stroke, transient ischaemic attack, and peripheral embolism in other trials (see *Table 1*). The definition of stroke across all trials was typically an acute focal neurological event that is positive on neuroimaging, or lasting greater than 24 h without neuroimaging. One trial mandated magnetic resonance imaging (MRI) as the imaging modality whereas the remainder allowed computed tomography (CT) or MRI. Two trials used the Amplatzer device, one the Gore sepal occluder, one the Starflex device (which is no longer available) and one trial used multiple device types. In terms of medical therapy, one trial left this open to physician discretion (specifying at least one agent); one allowed warfarin, aspirin, or both; and the remaining three trials allowed combinations of aspirin, clopidogrel, dipyridamole, or anticoagulation (see *Table 1*).

**Table 1:** Characteristics of included studies

Author	Study acronym	Year	Region	*n*	Mean age^a^	Follow-up^b^	Entry criteria	Intervention^c^	Medical therapy	Primary efficacy outcome	Safety outcomes	Definition of key clinical events
Mas *et al.*^10^	CLOSE	2017	France, Germany	473	42.9 (±10.1)	5.3 (±2.0)	Ischaemic stroke within previous 6 months. No identifiable cause except PFO with either ASA or large shunt. (ASA: 10 mm septum primum excursion. Large shunt: >30 microbubbles in LA within three cardiac cycles of RA opacification)	Dual antiplatelet therapy (aspirin with clopidogrel) for three months, followed by single antiplatelet therapy for the remainder of the trial (aspirin, clopidogrel, or aspirin with dipyridamole) Amplatzer PFO occluder (AGA Medical) Intrasept PFO occluder (Cardia) Premere (St Jude Medical) STARflex septal occluder system (NMT Medical) Amplatzer cribriform occluder (AGA Medical) Figulla Flex II PFO occluder (Occlutech, Inc.) Atriasept II occluder (Cardia) Amplatzer ASD occluder (AGA Medical) Figulla Flex II UNI occluder (Occlutech) Gore septal occluder (Gore Medical) Figulla Flex II ASD occluder (Occlutech)	Antiplatelet group Aspirin Clopidogrel Aspirin with dipyridamole Anticoagulation group Vitamin K antagonist Direct oral anticoagulant	Occurrence of fatal or non-fatal stroke	Major or fatal procedural or hemorrhagic complications	Ischaemic stroke: sudden onset focal neurological symptoms with the presence of cerebral infarction on CT/MRI regardless of duration or without imaging if >24 h duration.
Furlan *et al.*^6^	CLOSURE	2012	USA, Canada	909	46.3 (±9.6)	2	Ischaemic stroke or TIA within previous 6 months. No identifiable cause except PFO.	Dual antiplatelet therapy (aspirin with clopidogrel) for six months, followed by single antiplatelet for remainder of trial (aspirin) STARflex septal occluder system (NMT Medical)	Warfarin, aspirin or both	Composite of stroke/TIA in 2 years, all-cause mortality within 30 days, death from neurologic cause from 31 days to 2 years	Major bleeding	Stroke: Acute focal neurological event that is MRI positive (or if >24 h duration without imaging). TIA: sudden focal neurological event lasting >10 min without acute ischaemic brain injury on DWMR.
Meier *et al.*^4^	PC	2013	Europe, Canada, Brazil, Australia	414	44.3 (±10.2)	4.1	Ischaemic stroke, TIA, or a peripheral thrombo-embolic event. No identifiable cause except PFO.	Dual antiplatelet therapy (aspirin for at least 5 months and either ticlopidine or clopidogrel for 1–6 months) Amplatzer PFO occluder (AGA Medical)	Physician discretion (at least one agent)	Composite of death, non-fatal stroke, TIA, and peripheral embolism	Arrhythmias, device problems, bleeding	Non-fatal stroke: any neurologic deficit lasting for >24 h typically with CT/MRI documentation TIA: temporary neurologic deficit presumably due to reduced blood flow in a particular cerebral artery resolving in <24 h Peripheral Embolism: any non-brain end-organ ischaemia caused by reduced blood flow in a particular artery with imaging evidence (duplex, CT, or MRI)
Søndergaard *et al.*^11^	REDUCE	2017	USA, Canada, Denmark, Finland, Norway, Sweden, UK	664	45.4 (±9.3)	3.2	Ischaemic stroke within previous 6 months. No identifiable cause except PFO.	Aspirin, clopidogrel, or aspirin with dipyridamole for duration of trial Helex Septal Occluder (HELEX; W.L. Gore and Associates) Cardioform Septal Occluder (GSO; W.L. Gore and Associates)	Aspirin, clopidogrel or aspirin with dipyridamole for duration of trial	Freedom from clinical evidence of an ischaemic stroke for 24 months, new brain infarction (composite of clinical ischaemic stroke or silent brain infarction detected by presence of MRI changes)	Adverse events as classified by local investigators	Ischaemic stroke: sudden onset focal neurological symptoms with the presence of cerebral infarction on MRI/CT regardless of duration or without imaging if >24 h duration. New brain infarction: presence of at least one new hyperintense lesion at least 3 mm in diameter on T_2_ weighted MRI between the screening MRI and the 24 month MRI, as determined by the MRI core laboratory
Saver *et al.*^12^	RESPECT	2017	USA, Canada	980	45.9 (±9.9)	5.9	Ischaemic stroke within previous 9 months. No identifiable cause except PFO.	Dual antiplatelet therapy (aspirin with clopidogrel) for one month followed by 5 months of aspirin alone with further therapy at investigator’s discretion. Amplatzer PFO occluder (AGA Medical)	Aspirin, warfarin, clopidogrel, aspirin with dipyridamole (Aspirin with clopidogrel allowed until 2006)	Composite of recurrent non-fatal ischaemic stroke, fatal ischaemic stroke, or early death (30 days after procedure, 45 days after randomization)	Adverse events as classified by adjudicated by data and safety monitoring board	Ischaemic stroke: sudden onset focal neurological symptoms with the presence of cerebral infarction on CT/MRI regardless of duration or without imaging if >24 h duration.

CT, computed tomography; DWMR, diffusion weighted magnetic resonance; MRI, magnetic resonance imaging; PFO, patent foramen ovale; TIA, transient ischaemic attack.

a Mean age in years (±SD); value for intervention group provided where values differ between intervention and comparison groups.

b Follow-up in years (mean ± SD, where provided, except REDUCE & RESPECT, where medians are provided); value for intervention group provided where values differ between intervention and comparison groups.

c Antiplatelet regime for intervention group with device(s) used in italics.

Trial quality was assessed using the Cochrane risk of bias tool and is shown in *Table 2*. All five trials were conducted with bias-resistant features such as randomization and reporting of all pre-specified outcomes. Each trial was specified to be open-label so there was no blinding of patients. Three trials (CLOSE, CLOSURE, and REDUCE) did not specify independent, blinded adjudication of clinical events and, as such, were judged to be of intermediate quality since blinded assessment of outcomes is the most important potential bias in trials where the primary endpoint is often a physician-determined clinical syndrome. The remaining two trials (PC and RESPECT) specifically reported independent adjudication of clinical events by assessors unaware of treatment allocation. They were judged to be of high quality.

**Table 2:** Risk of bias assessment

Trial	Random sequence generation	Allocation concealment	Blinding of participants and personnel	Blinding of outcome assessment	Incomplete outcome data	Selective reporting	Overall Quality
CLOSE	Low risk ‘Dedicated Web-based software’—permuted blocks with variable block size	Unclear ‘Dedicated Web-based software’	High risk Un-blinded	High risk Specifically stated that study neurologists were aware of treatment allocation	Low risk 1 patient withdrew consent after randomization (no data according to French law) 2 patients in control arm lost to follow-up. 3 patients in intervention arm did not receive intervention (2 refused, 1 no PFO). 17 patients in intervention arm discontinued (7 patient choices, 7 medical decisions, 3 haemorrhagic complications). 10 patients in intervention arm discontinued treatment (5 patient choices, 4 medical decisions, 1 haemorrhagic complication).	Low risk All endpoints on CT.gov reported	Intermediate A well-conducted open-label trial but the absence of independent, blinded adjudication of clinical events reduces the quality of this trial.
CLOSURE	Unclear Not specified	Unclear Not specified	High risk Un-blinded	High risk Not specified (in an open-label trial)	Low risk PFO: 3 did not receive treatment (2 refused, 1 no PFO). 17 discontinued antiplatelet (7 patient choices, 7 medical decisions, 3 haemorrhagic complications). 1 PFO and ASD at intervention. All included in ITT. Antiplatelet 2 lost to follow-up, 10 discontinued (5 patient choices, 4 medical decisions, 1 haemorrhagic complication) all included in ITT	Low risk All endpoints on CT.gov reported	Intermediate A well-conducted open-label trial but the absence of independent, blinded adjudication of clinical events reduces the quality of this trial.
PC	Unclear ‘Web-based system’	Unclear ‘Web-based system’	High risk Un-blinded for participants and some personnel	Low risk Independent adjudicators of events were unaware of assignment	Low risk 17 closure patients and 11 medical therapy patients withdrew, 24 and 31 lost to follow-up.	Low risk All endpoints on CT.gov reported	High A well-conducted open-label trial with independent, blinded adjudication of clinical events.
REDUCE	Unclear Not specified	Unclear Not specified	High risk Un-blinded	Unclear Specifically stated that study neurologists were aware of treatment allocation	Low risk Closure: 15 LTFU, 9 withdrew consent, 3 investigator withdrew, 1 death Medical therapy: 7 LTFU, 15 withdrew consent, 3 investigators withdrew.	Low risk All endpoints on CT.gov reported	Intermediate A well-conducted open-label trial but the absence of independent, blinded adjudication of clinical events reduces the quality of this trial.
RESPECT	Unclear Not specified	Unclear Not specified	High risk Un-blinded	Low risk Independent adjudicators of events were unaware of assignment	High risk Closure: 56 LTFU, 19 withdrew consent, 1 subject withdrew 1 investigator requested withdrawal. 32 did not attempt device despite assignment to device: 4 LTFU, 10 withdrew consent, 4 subject withdrawal. 2 investigators requested. Medical therapy: 67 LTFU, 78 withdrew consent mainly to seek PFO closure, 4 investigator requested	Low risk All endpoints on CT.gov reported	High A well-conducted open-label trial with independent, blinded adjudication of clinical events.

ITT, intention to treat; LTFU, lost to follow-up.

### Efficacy of closure vs. medical therapy

**Figure 2:**
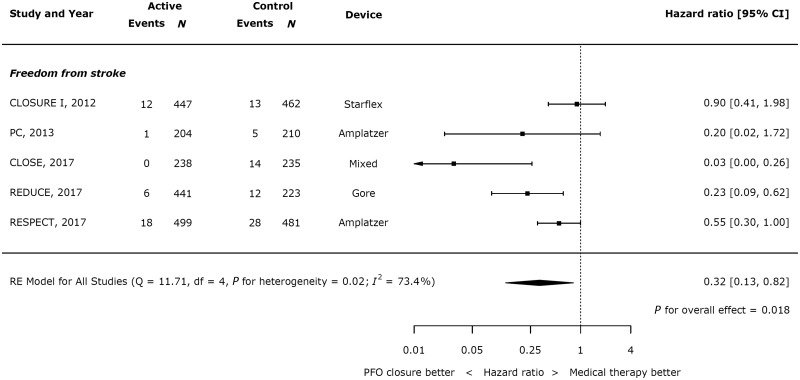
Effect of device closure on recurrent stroke.

Closure of PFO resulted in a significant reduction in recurrent stroke (*Figure 2*; HR 0.32 95% CI 0.13–0.82; *P* = 0.018), though with significant heterogeneity (*I*^2^ = 73.4%). Across all trials, 37 of 1829 patients had a recurrence of stroke in the active arms, compared with 72 of 1611 in the control arms. Overall, the annual weighted risk of recurrent stroke was low in both in the closure (0.61%) and the medical therapy (1.17%) group.

### Safety of closure vs. medical therapy

**Figure 3:**
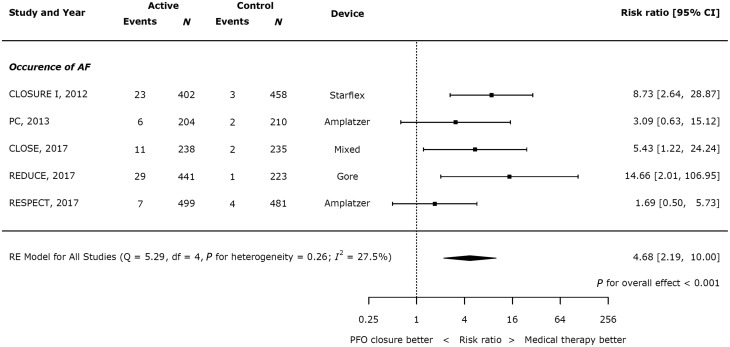
Effect of device closure on atrial fibrillation.

Device closure significantly increased the risk of AF (*Figure 3*; risk ratio (RR) 4.68, 95% CI 2.19–10.00, *P*<0.001, heterogeneity *I*^2^ = 27.5%). Across all trials, 76 of 1784 patients had a recurrence of stroke in the active arms, compared with 12 in the control arms. Overall, the annual weighted risk of AF was low (1.38% per year in the device arm and 0.21% per year in the control arm).

**Figure 4:**
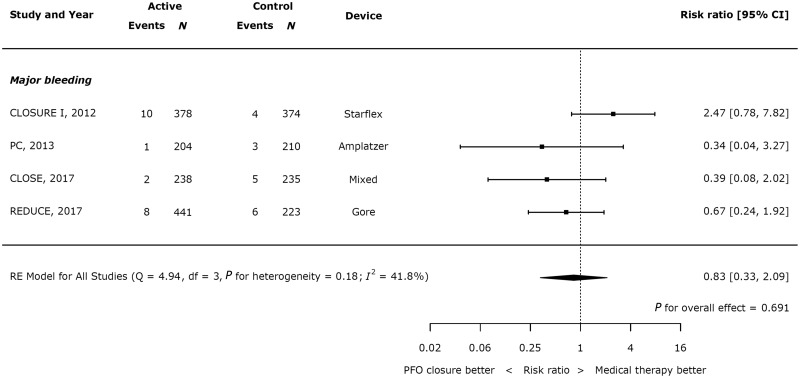
Effect of device closure on major bleeding.

Across the four studies which reported major bleeding in both the device and control arms, there was no significant difference between the groups (*Figure 4*, RR 0.83, 95% CI 0.33–2.09; *P* = 0.691, heterogeneity *I*^2^ = 41.8%).

Procedural-related events were low across the five trials, occurring in 3.2% of patients in CLOSURE-1, 5.9% in CLOSE, 2.5% in REDUCE, 1.5% in PC, and 2.4% of RESPECT.

### Impact of shunt size and atrial septal aneurysm

Three trials reported outcomes stratified by shunt size and presence of an atrial septal aneurysm (CLOSURE I, PC, and RESPECT). The CLOSE trial only enrolled patients with either a large shunt or an atrial septal defect (ASD) and therefore there was no appropriate comparator group for either variable.

**Figure 5:**
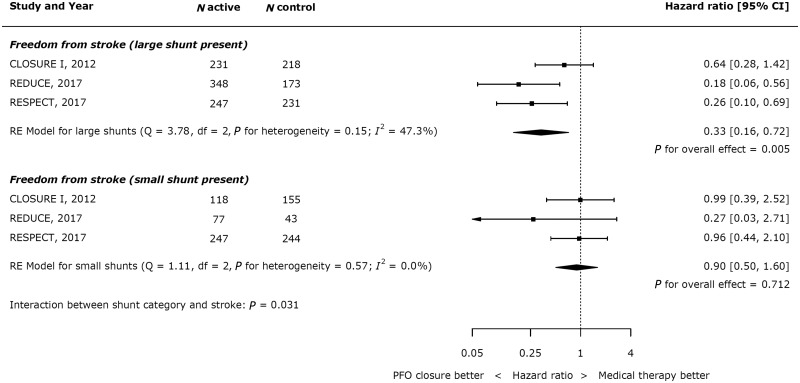
Impact of shunt size on effect of device closure on recurrent stroke.

In patients with a large shunt, PFO closure was associated with a significant reduction in stroke (*Figure 5*; HR 0.33, 95% CI 0.16–0.72; *P* = 0.005), whilst there was no significant reduction in stroke in patients with a small shunt (HR 0.90, 95% CI 0.50–1.60; *P* = 0.712). Across all trials, 7 of 478 patients with a large shunt had a recurrent stroke in the active arm, compared with 13 of 275 patients with a large shunt in the control arms. In patients with large shunts the annual risk of stroke was 0.53% in patients undergoing PFO closure and 1.56% in those treated medically. There was a statistically significant p value for interaction between shunt category and the risk of recurrent stroke (*P* = 0.031).

**Figure 6:**
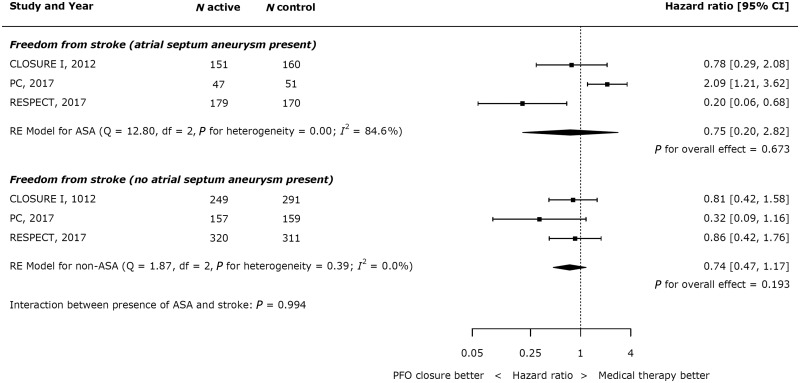
Impact of atrial septal aneurysm on effect of device closure on recurrent stroke.

There was no effect from the presence or absence of an atrial septal aneurysm on outcomes (see *Figure 6*).

### Sensitivity analysis

A sensitivity analysis excluding the CLOSURE-1 trial (which used the Starflex device which is no longer used in clinical practice) showed similar results to the primary analyses for efficacy and safety (see Supplementary material online, *Figures S1andS2*).

We also performed a full jack-knife analysis excluding each trial in turn, for both the primary efficacy and primary safety outcomes. The results are shown in Supplementary material online, *Figures S3–S10*.

## Discussion

In this study, we have shown that: (i) the risk of recurrent stroke is low in RCTs for patients with PFO who have suffered a cryptogenic stroke (annual risk of 1.16% in patients treated with medical therapy in trials); (ii) this risk can be reduced by two-thirds by device closure of a PFO in addition to medical therapy (HR 0.32 with device closure, 95% CI 0.13–0.82; *P* = 0.018); (iii) the benefit from PFO closure depends on shunt size (*P* = 0.031), with patients with large shunts benefitting from device closure (HR 0.33, 95% CI 0.16–0.72; *P* = 0.005), in contrast to those with small shunts (HR 0.90, 95% CI 0.50–1.60; *P* = 0.712); (iv) there is no impact of the presence or absence of an aneurysmal atrial septum on outcomes with device closure; and (v) AF is rare but significantly increased with device closure vs. medical therapy (annual risk 1.38% in device arm, RR 4.68, 95% CI 2.19–10.00, *P*<0.001 with device closure).

### Superiority of device closure to medical therapy

Previous RCTs and meta-analyses have failed to show the superiority of device closure to medical therapy for the prevention of stroke in patients with a PFO. With the publication of two new RCTs, we have robust data to show that device closure is associated with a significant reduction in recurrent stroke. Furthermore, we now have data to support PFO closure with multiple device types (previously the only positive data was for the Amplatzer device). Our primary analysis, which included all RCTs published in this field, showed clear superiority of PFO closure; this was reinforced in a sensitivity analysis excluding the CLOSURE-1 trial which used the now defunct Starflex device, where the margins of superiority for stroke prevention were greater and the margins of inferiority for risk of AF were smaller.

The significance of PFO in the aetiology of cryptogenic stroke has been debated.^19^^,^^20^ This analysis demonstrates that interventional strategies can significantly reduce risk of recurrent stroke and supports the underlying mechanistic plausibility for causation of stroke via paradoxical embolism. With this combination of factors, we can now state that, in selected patients with a cryptogenic stroke and a PFO, device closure reduces future events.

### The benefit from patent foramen ovale closure depends on shunt size

Given the suspicion that larger shunt sizes could be associated with a higher risk of paradoxical flow and hence stroke, several trials in this meta-analysis provided HRs stratified by shunt size. This meta-analysis reveals a statistically significant interaction between shunt size and the reduction in hazard of stroke (*P* = 0.031). Therefore, shunt size should be considered in our clinical decision-making when evaluating patients for potential device closure. Conversely, this analysis did not show a statistically significant interaction between the presence of an atrial septal aneurysm and the reduction in the hazard of stroke (*P* = 0.994). This may be because in patients with an aneurysmal septum it is harder to achieve complete closure of the defect with device therapy. These associations require further investigation.

### Magnitude of benefit

For all patients included in this analysis, the absolute risk reduction per year with device closure over medical therapy is 0.56, translating to a number needed to treat of 178 when dichotomized over this short-term basis. When considering patients with large shunts, the number needed to treat to prevent one stroke in 1 year is 96.

This may appear a large number to recommend adoption of this procedure, but PFO closure is a single intervention with a low rate of complications. Furthermore, patients consider a stroke a worse outcome than death,^21^ and the patients enrolled in these trials—and who should be considered for device therapy in clinical practice—will be young and all have made a good recovery from their index stroke. Finally, this number needed to treat quoted is per year. Given the Kaplan–Meier plots in the analysed trials demonstrate proportional hazards it is likely valid to assume the reduced risk of stroke will continue beyond the follow-up times analysed in the trials. Therefore, the number needed to treat at a single time-point does not reflect the large number of disease-free life years that will be gained from device closure.

### Guidelines

Current guideline recommendations are against device closure for patients with a PFO and cryptogenic stroke. The most contemporary guidance, from the American Academy of Neurology in 2016,^22^ states that ‘clinicians should not routinely offer percutaneous PFO closure to patients with cryptogenic ischaemic stroke outside of a research setting’. Joint guidance from the American Heart Association and American Stroke Association in 2014^23^ also did not endorse device closure, while The American College of Chest Physicians gave a Class II recommendation^24^ only in patients who experience recurrent events despite aspirin therapy. All existing guideline recommendations are summarized in *Table 3*.

**Table 3:** Guideline recommendations for patent foramen ovale closure

Guideline	Year	Recommendation
European Society of Cardiology^27^	2010	In the case of documented systemic embolism probably caused by paradoxical embolism, isolated device closure of ASD/PFO should be considered (*Class IIa; Level of Evidence C*)
American College of Chest Physicians (ACCP)^25^	2012	In patients with cryptogenic stroke and PFO or atrial septal aneurysm, who experience recurrent events despite aspirin therapy, we suggest treatment with VKA therapy (target INR 2.5; range 2.0–3.0) and consideration of device closure over aspirin therapy (Grade 2C) In patients with cryptogenic stroke and PFO, with evidence of DVT, we recommend VKA therapy for 3 months (target INR 2.5; range 2.0–3.0) (Grade 1B) and consideration of device closure over no VKA therapy or aspirin therapy (Grade 2C)
National Institute for Health and Care Excellence (NICE)^28^	2013	Evidence on the safety of percutaneous closure of patent foramen ovale to prevent recurrent cerebral embolic events shows serious but infrequent complications. Evidence on its efficacy is adequate. Therefore this procedure may be used with normal arrangements for clinical governance, consent, and audit.
American Heart Association/American Stroke Association (AHA/ASA)^24^	2014	For patients with a cryptogenic ischaemic stroke or TIA and a PFO without evidence for DVT, available data do not support a benefit for PFO closure (*Class III; Level of Evidence A*). In the setting of PFO and DVT, PFO closure by a transcatheter device might be considered, depending on the risk of recurrent DVT (*Class IIb; Level of Evidence C*).
American Academy of Neurology (AAN)^23^	2016	Clinicians should not routinely offer percutaneous PFO closure to patients with cryptogenic ischaemic stroke outside of a research setting (Level R). For recurrent strokes despite adequate medical therapy with no other mechanism identified, clinicians may offer the AMPLATZER PFO Occluder if it is available (Level C)

DVT, deep vein thrombosis; INR, international normalized ratio; TIA, transient ischaemic attack; VKA, vitamin K antagonist.

The results of this meta-analysis should inform an update in the guideline recommendations and suggest that in patients with large shunts the recommendation for device closure is strong.

### Implications for clinical research

In 2009, the ACC/AHA and ASA published a statement document^25^^,^^26^ calling for ‘the completion of randomized clinical trials’ studying device closure of PFOs for secondary stroke prevention. The context of this statement was the slow recruitment and high drop-out rates observed in RCTs. Factors underpinning this may include a reluctance from clinicians to randomize their patients owing to belief in the effectiveness of device closure; young patients who had suffered a stroke also felt committed to choosing their own treatment rather than having it left to chance. This led to increasing off-label use of closure devices and the aforementioned difficulty in completing RCTs in a timely manner, with increased drop-out rates observed in the medical therapy groups compared to the device groups. Furthermore, the expected event rates in the medical therapy arm used to power earlier studies were generally double the event rates observed in the trials. This combination of factors perhaps goes some way towards explaining the negative results reported in earlier RCTs of PFO closure. During the time-windows of all RCTs reported in this analysis, 20 times as many patients underwent device closure outside a trial setting than within it.

If clinicians and patients were more willing to participate in randomized trials, the answer shown by the more contemporary RCTs—and this meta-analysis—may have been available many years ago. This would have led to an earlier adoption of an efficacious herapy, with consequent benefits to patients. Furthermore, it could have prevented expending research resources answering a question which might have been resolved much earlier.

### Limitations

Two recent abbreviated analyses have been published as Letters showing benefit of PFO closure.^26^^,^^27^ Our analysis provides additional insights in several regards. First, we have used HR rather than RR as our primary endpoint, which is more appropriate for time-to-event data. Secondly, we have performed sensitivity analyses examining the effect of each individual trial on both the overall results and the heterogeneity. Thirdly, we have performed subgroup analyses for the effect of large shunts and the presence of an aneurysmal atrial septum. Finally, we have reported additional safety endpoints for bleeding and device complications.

We could only report the available data, and cannot account for unpublished trials. There were differences in methodology and reporting across the studies. Follow-up duration varied across the studies, and there were differences in the entry criteria and primary endpoints. The definitions of clinical events and subgroups (including shunt size) were not uniform across trials, but this problem is common to all meta-analyses. Clinical researchers should endeavour to standardize definitions of events and subgroups across trials to better permit synthesis of their results.

The variations in endpoint definitions likely contributed to the heterogeneity observed in results for the primary outcome. REDUCE and RESPECT shared eligibility criteria with regard to thromboembolism: including patients with cryptogenic ischaemic strokes with a requirement for CT or MR infarct if the symptoms lasted for less than 24 h and explicitly excluding lacunar strokes. Their definitions of the stroke endpoint were also identical to each other: stroke with requirement for CT or MR infarct if symptoms lasted less than 24 h. The inclusion criteria and endpoint stroke definition for CLOSE were similar, except there was no explicit mention of excluding lacunar strokes. CLOSURE included the same patients but also included patients whose symptoms lasted less than 24 h without evidence of infarct but the stroke endpoint definition excluded such events (transient ischaemic attack was only included if there was an associated radiological infarct, as for CLOSE, REDUCE, and RESPECT). Lacunar strokes were not explicitly excluded. PC included the same population as CLOSE, REDUCE, and RESPECT but also included patients with peripheral thromboembolism and did not explicitly exclude lacunar strokes. However, the stroke endpoint in PC excluded any event lasting less than 24 h, regardless of the presence of radiological infarct (these were considered to be TIAs). As such, the more lenient criteria of CLOSURE may have resulted in a lower risk status of patients in this trial, including TIAs without radiological infract and potentially including lacunar strokes. The stricter stroke definition in PC may have reduced its event rate for this endpoint.

Furthermore, there was variation in the type of device used for PFO closure, and one included trial used a device that is no longer in clinical use; our sensitivity analysis, however, showed the results were similar whether or not we included this trial.

Medical therapy was also not uniform across studies, with some using antiplatelet therapy (which could be aspirin, dipyridamole, or clopidogrel in various combinations), and others anticoagulant therapy. These differences may explain the significant heterogeneity seen between trials assessing the hazard of stroke following PFO closure (*I*^2^ = 73.4%, *P* = 0.02).

During stepwise omission of each trial, heterogeneity reduced with the omission of CLOSE (46.8%), CLOSURE I (65.3%), and RESPECT (74.6%), but increased with the omission of PC (82.3%) and REDUCE (81.2%). This heterogeneity was most heavily influenced by the CLOSE trial, which was the most strongly in favour of device closure. Potential reasons for this are that only patients with a large shunt or aneurysmal atrial septum were included; the requirement of confirmation of cerebral infarction on neuroimaging as part of the definition of stroke; or the fact that enrolled patients had a very low burden of vascular risk factors. Importantly, in the sensitivity analysis excluding the CLOSE trial there was still a statistically significant benefit of device closure (HR 0.48, 95% CI 0.25–0.91; *P* = 0.025).

Future trials may wish to investigate the role of new oral anticoagulants in this setting rather than antiplatelet therapy. However, we note that the recent NAVIGATE-ESUS trial, comparing aspirin to rivaroxaban for secondary prevention of stroke and systemic embolism, was stopped early due to futility: there was no difference in efficacy between the two groups, and an excess of bleeding with rivaroxaban. Therefore, antiplatelet therapy is currently the more appropriate comparator to device therapy for these patients.

## Conclusions

In selected patients with cryptogenic stroke, PFO closure is superior to medical therapy for the prevention of further stroke: this is particularly true for patients with moderate-to-large shunts. Guidelines should be updated to reflect this.

## Supplementary material

Supplementary material is available at *European Heart Journal* online.

## Supplementary Material

Online Appendix
