# Supplementary material for: Patent foramen ovale closure vs. medical therapy for cryptogenic stroke: a meta-analysis of randomized controlled trials
Source: Eur Heart J. 2018 Mar 24;39(18):1638–49. doi: 10.1093/eurheartj/ehy121 (PMC5946888; doi:10.1093/eurheartj/ehy121)
Supplement: Online Appendix [file ehy121_online_appendix.docx]

Online Figure 1. Sensitivity analysis excluding the CLOSURE-1 trial, for the primary efficacy endpoint

Online Figure 2. Sensitivity analysis excluding the CLOSURE-1 trial, for the primary safety endpoint.

Online Figure 3. Sensitivity analysis excluding the CLOSE trial, for the primary efficacy endpoint

Online Figure 4. Sensitivity analysis excluding the CLOSE trial, for the primary safety endpoint

Online Figure 5. Sensitivity analysis excluding the PC trial, for the primary efficacy endpoint

Online Figure 6. Sensitivity analysis excluding the PC trial, for the primary safety endpoint

Online Figure 7. Sensitivity analysis excluding the REDUCE trial, for the primary efficacy endpoint

Online Figure 8. Sensitivity analysis excluding the REDUCE trial, for the primary safety endpoint

Online Figure 9. Sensitivity analysis excluding the RESPECT trial, for the primary efficacy endpoint

Online Figure 10. Sensitivity analysis excluding the RESPECT trial, for the primary safety endpoint
